# Validation of PhenX measures in the personalized medicine research project for use in gene/environment studies

**DOI:** 10.1186/1755-8794-7-3

**Published:** 2014-01-14

**Authors:** Catherine A McCarty, Richard Berg, Carla M Rottscheit, Carol J Waudby, Terrie Kitchner, Murray Brilliant, Marylyn D Ritchie

**Affiliations:** 1 Division of Research, Essentia Institute of Rural Health, 502 East Second Street, Duluth, MN 55805, USA; 2 Marshfield Clinic Research Foundation, Marshfield, USA; 3 Pennsylvania State University, University Park, USA

## Abstract

**Background:**

The purpose of this paper is to describe the data collection efforts and validation of PhenX measures in the Personalized Medicine Research Project (PMRP) cohort.

**Methods:**

Thirty-six measures were chosen from the PhenX Toolkit within the following domains: demographics; anthropometrics; alcohol, tobacco and other substances; cardiovascular; environmental exposures; cancer; psychiatric; neurology; and physical activity and physical fitness. Eligibility criteria for the current study included: living PMRP subjects with known addresses who consented to future contact and were not currently living in a nursing home, available GWAS data from eMERGE I for subjects where age-related cataract, HDL, dementia and resistant hypertension were the primary phenotypes, thus biasing the sample to the older PMRP participants. The questionnaires were mailed twice. Data from the PhenX measures were compared with information from PMRP questionnaires and data from Marshfield Clinic electronic medical records.

**Results:**

Completed PhenX questionnaires were returned by 2271 subjects for a final response rate of 70%. The mean age reported on the PhenX questionnaire (73.1 years) was greater than the PMRP questionnaire (64.8 years) because the data were collected at different time points. The mean self-reported weight, and subsequently calculated BMI, were less on the PhenX survey than the measured values at the time of enrollment into PMRP (PhenX means 173.5 pounds and BMI 28.2 kg/m^2^ versus PMRP 182.9 pounds and BMI 29.6 kg/m^2^). There was 95.3% agreement between the two questionnaires about having ever smoked at least 100 cigarettes. 139 (6.2%) of subjects indicated on the PhenX questionnaire that they had been told they had a stroke. Of them, only 15 (10.8%) had no electronic indication of a prior stroke or TIA. All of the age-and gender-specific 95% confidence limits around point estimates for major depressive episodes overlap and show that 31% of women aged 50–64 reported symptoms associated with a major depressive episode.

**Conclusions:**

The approach employed resulted in a high response rate and valuable data for future gene/environment analyses. These results and high response rate highlight the utility of the PhenX Toolkit to collect valid phenotypic data that can be shared across groups to facilitate gene/environment studies.

## Background

The National Human Genome Research Institute funded the development of consensus measures for Phenotypes and eXposures (PhenX) [1,2]. The goal of PhenX was to develop 15 measures for 21 different phenotypic domains. Data collection worksheets are available through the PhenX Toolkit (http://www.phenxtoolkit.org), with the hope that broad acceptance and use of the PhenX measures will allow for cross-study comparisons and improve the statistical power for gene/environment analyses in the context of genome-wide association studies (GWAS). PhenX measures were selected by working groups of domain experts using a consensus process that included input from the scientific community.

The eMERGE network (http://www.gwas.net), also funded by the National Human Genome Research Institute, is a national consortium formed to develop, disseminate, and apply approaches to research that combine DNA biorepositories with electronic medical record (EMR) systems for large-scale, high-throughput genetic research [3]. The Marshfield Clinic Personalized Medicine Research Project (PMRP) [4] was one of the five initial eMERGE sites, with cataract, HDL and diabetic retinopathy as the primary phenotypic outcomes.

An administrative supplement funded by NHGRI to the eMERGE grant allowed PMRP investigators to collect PhenX measures for subjects with available GWAS data from eMERGE. The PMRP team was one of seven sites to makeup the PhenX RISING network that was funded through administrative supplements to incorporate PhenX measures into existing population-based genomic studies (https://www.phenxtoolkit.org/index.php?pageLink=phenxrising). In total, the seven groups incorporated 76 PhenX measures, representing a quarter of the 295 measures present in the Toolkit as of July 2011. The measures encompass demographics, psychosocial risk factors, psychiatric assessments, and a variety of exposures. Each group is adding between 4 and 37 measures with five groups, including PMRP, adding more than 20 measures. In all, 55 of these 81 measures are shared by two or more groups providing common ground for future cross-study analysis.

The purpose of this paper is to describe the data collection efforts and validation of the PhenX measures in the PMRP cohort.

## Methods

The Marshfield Clinic Personalized Medicine Research Project (PMRP) is a population-based biorepository linked to the comprehensive electronic medical record of Marshfield Clinic, details of which have been published previously [4]. Self-administered questionnaire data are available for the cohort to facilitate gene/environment analyses, including the detailed Dietary History Questionnaire [5].

As part of the initial written informed consent to participate in PMRP, subjects were given the option to opt out of future contact. Less than 1% of subjects elected this option. Eligibility criteria for the current study included: living PMRP subjects with known addresses who consented to future contact and were not currently living in a nursing home. In addition, subjects were required to have available GWAS data from eMERGE I, where age-related cataract, HDL, dementia and resistant hypertension were the primary phenotypes [6], thus biasing the sample to the older PMRP participants.

The current study was reviewed and approved by the institutional review boards at Marshfield Clinic and Essentia Institute of Rural Health. The PhenX Toolkit (http://www.phenxtoolkit.org) was accessed to develop a self-administered questionnaire to include the 36 items listed in Table 1. Also listed in Table 1 are all data elements available for comparison with PMRP. Some of the PhenX measures were included because of the potential for gene/environment associations with age related cataract (smoking, alcohol, ultraviolet light exposure), some were included because data were available for validation by comparison with prior PMRP questionnaire data and medical history information (demographics, physical activity, family history of heart attack, history of stroke) and the rest were included because of the potential for future research and cross-site collaborations (hypomania/mania symptoms, hand dominance) within the PhenX RISING network funded through administrative supplements to collect PhenX measures. The time to complete the questionnaire ranged from 20 to 40 minutes in pre-testing, depending on how many questions were logical skips.

**Table 1:** **PhenX Toolkit measures employed in the current study and availability**/**comparability of PMRP data for validation**

**PhenX ID**	**PhenX measure name**	**Available PMRP data source for comparison**	**Comparability of measures (N/A for not applicable where there are no data for comparison)**
010101	Current age	EMR	Not identical but expect congruence because construct is same
010201	Birthplace	No data	N/A
010301	Birthplace of parents	No data	N/A
010401	Birthplace of grandparents	No data	N/A
010501	Ethnicity	Enrollment questionnaire (US Census question)	Fewer forced options were available on the PMRP questionnaire then the PhenX questionnaire based on expected responses prior to the “other/please specify” option. Construct is the same.
010601	Race	Enrollment questionnaire (US Census question)	Fewer forced options were available on the PMRP questionnaire then the PhenX questionnaire based on expected responses prior to the “other/please specify” option. Construct is the same.
010701	Gender	EMR	Not identical but expect congruence because construct is same
010801	Current address	EMR	Not identical but expect congruence because construct is same
010901	Current marital status	No data	N/A
011001	Current educational attainment	Questionnaire for subset post enrollment	Not identical but expect congruence because construct is same, fewer forced categories for lower levels of education in PMRP questionnaire based on expected responses
011301	Current employment status	Enrollment questionnaire (US Census question)	Similar construct, but not identical questions. PhenX includes 8 choices for current working status. PMRP questionnaire asked about being employed in the previous 5 years (yes/no)
011401	Household roster – relationships	No data	N/A
011501	Health insurance coverage	EMR	Not identical but expect congruence because construct is same
020602	Hand dominance (12 years and older)	No data	N/A
020704	Self-reported height	Measured at enrollment, EMR	Same construct, but expect some over-reporting based on literature
021502	Self-reported weight	Measured at enrollment, EMR	Same construct, but expect some under-reporting based on literature
030101	Alcohol – lifetime use	No data	N/A
030201	Alcohol – age of first use	No data	N/A
030301	Alcohol – 30-day frequency and quantity	Enrollment questionnaire	Same construct, but PhenX did not have forced category responses. PhenX had open-ended number of days and number of drinks per day. PMRP had forced categories of response (<1, 1–2, 3–4, 5–7 days per week and 1, 2, 3–4, 50 or more drinks per day)
030401	Alcohol – maximum drinks in 24 hours	No data	N/A
030501	Alcohol – lifetime abuse and dependence	No data	N/A
030602	Tobacco – smoking status (adult protocol)	Enrollment questionnaire	Identical questions
030702	Tobacco – age of initiation of use (adult protocol)	No data	N/A
030802	Tobacco – 30-day quantity and frequency (adult protocol)	Enrollment questionnaire	Identical questions
030902	Tobacco – age of offset of use (adult protocol)	No data	N/A
031001	Tobacco – nicotine dependence	No data	N/A
040101	Family history of heart attack	Family history of heart or stroke from enrollment questionnaire	Similar construct but definition of relatives is different and two health outcomes were included in the PMRP questionnaire
060101	Characteristics of current residence	No data	N/A
060701	Current environmental tobacco smoke exposure	No data	N/A
060801	Sibship/birth order	No data	N/A
061301	Ultraviolet light exposure	No data	N/A
070301	Passive smoke exposure	No data	N/A
120402	Hypomania/mania symptoms - adult	No data	N/A
120502	Depression – adult	No data	N/A
130301	History of stroke – ischemic infarction and hemorrhage	EMR	Similar construct but difficult to determine if people completing PhenX questionnaire understand medical terms
150901	Total physical activity screener	No comparable data	N/A

The 32-page self-administered questionnaire was mailed to all eligible subjects with a cover letter and return address envelope. A second mailing was employed to increase the response rate. Subjects were offered $10 for their time to complete the questionnaire. The mailings occurred at the end of 2011 and beginning months of 2012. The majority of PMRP participants were enrolled between September 2002 and April 2004 so there is a considerable time lag between completion of questionnaires.

PhenX survey data were entered and merged with prior PMRP questionnaire information and data about prior stroke from clinical diagnoses in the Marshfield Clinic electronic medical record. Analyses in this report include standard descriptive statistics and approximate confidence limits. For validation purposes, the clinical diagnoses and measurements from electronic medical record were considered to be the gold standard when it was used for comparison. The signed-ranks test was used to compare PhenX self-reported weight and BMI with measurements at PMRP enrollment, simple kappa statistics and 95% CL were calculated for nominal categories and Fleiss-Cohen weighted kappas and 95% CL for ordinal classifications as appropriate. A p-value <0.05 was considered statistically significant.

## Results

Questionnaires were mailed to 3344 PMRP participants with GWAS data. The denominator decreased to 3246 after participants were removed for eligibility reasons (no known address, current nursing home residence, deceased). Completed questionnaires were returned by 2271 subjects for a final response rate of 70%. Upon comparing age and gender responses with Marshfield Clinic EMR data, it was determined that two of the respondents were the spouses of intended respondents who had participated in the PMRP biobank but for whom GWAS data were not available. Fifty-nine percent of the respondents were female, reflecting a similar response rate by gender (Table 2). The vast majority of the study population reported being White (96.2%) and of non-Hispanic (93.3%) ethnicity. The gender and race/ethnicity of the respondents to the PhenX survey is nearly identical to the original PMRP cohort, which is similar to the general population of central Wisconsin, other than an under-representation from men who were less likely to participate initially in the PMRP biobank [4].

**Table 2:** Demographic and anthropometric data as reported on PhenX survey and at PMRP enrollment

	**PhenX gender**									
**PMRP**		**Female**		**Male**		**Unknown**		**Combined**		
**Gender**		**N (%)**		**N (%)**		**N (%)**		**N (%)**		
Female		**1344 (59.2)**		0 (0)		17 (0.7)		1361 (59.9)		
Male		0 (0)		**898 (39.5)**		12 (0.5)		910 (40.1)		
Combined		1344 (59.2)		898 (39.5)		29 (1.3)		2271 (100.0)		
Kappa = 1.0 (95% CL=1.0, 1.0)										
	**PhenX Race**									
**PMRP race**	**White only**		**White+Other**		**Other only**		**Unknown**		**Combined**	
	**N (%)**		**N (%)**		**N (%)**		**N (%)**		**N (%)**	
White Only	**2177 (95.9)**		1 (0.0)		3 (0.1)		67 (3.0)		2248 (99.0)	
White+Other	8 (0.4)		**4 (0.2)**		2 (0.1)		0 (0)		6 (0.3)	
Other Only	2 (0.1)		0 (00		**4 (0.2)**		0 (0)		3 (0.1)	
Unknown	2 (0.1)		0 (0)		1 (0.0)		**0 (0)**		3 (0.1)	
Combined	2189 (96.4)		5 (0.2)		10 (0.4)		67 (3.0)		2271 (100.0)	
Kappa = 0.605 (95% CL=0.387, 0.823)										
	**PhenX ethnicity**									
**PMRP ethnicity**		**Hispanic/Latino**		**Not hispanic**		**Unknown**		**Combined**		
		**N (%)**		**N (%)**		**N (%)**		**N (%)**		
Hispanic/Latino		**4 (0.2)**		1 (0.0)		0 (0)		5 (0.2)		
Not Hispanic		8 (0.4)		**2118 (93.3)**		140 (6.2)		2266 (99.8)		
Combined		12 (0.5)		2119 (93.3)		140 (6.2)		2271 (100.0)		
Kappa = 0.469 (95% CL=0.175, 0.763)										
	**PhenX survey**					**PMRP questionnaire**				
	**N**	**Mean**	**S.D.**	**Min.**	**Max.**	**N**	**Mean**	**S.D.**	**Min.**	**Max.**
Age (years)	2271	7301	10.1	54.	101.7	2271	64.8	10.2	45.7	93.2
Weight (lbs)	2172	173.5	39.5	58.0	399.0	2172	182.9	39.3	76.0	350.0
Height (in)	2146	65.8	4.1	38.0	105.0	2146	65.8	3.7	48.0	77.0
BMI (kg/m^2^)	2109	28.2	5.8	10.3	75.4	2109	29.6	5.6	17.4	61.2

Agreement noted in bold.

There was good agreement between the PhenX Toolkit questions and the PMRP questionnaire on demographics. The mean age at completion of the PhenX questionnaire (73.1 years) was greater than the PMRP age at enrollment from the EMR (64.8 years) because the data were collected at different time points. The mean self-reported weight, and subsequently calculated BMI, were significantly less on the PhenX survey than the measured values at the time of enrollment into PMRP (PhenX means 173.5 pounds and BMI 28.2 versus PMRP 182.9 pounds and BMI 29.6, each p < 0.001).

The smoking and alcohol questions are far more detailed in the PhenX measures than the PMRP questionnaire. Table 3 present a comparison of responses to identical smoking questions from the two sources, queried on average eight years apart. There was 95.3% agreement between the two questionnaires about having ever smoked at least 100 cigarettes. The agreement between the two questionnaires for frequency of current smoking was also 95.3%. Kappa statistics reflect the lower agreement for current smoking than ever smoked (0.673 versus 0.905). The agreement for self-reported alcohol intake was not as strong as for smoking and lower for usual drinks per day in comparison with drinking in the past 30 days (69.6% agreement for drinking in the past 30 days, Table 4, kappa = 0.666; and 73.9% for usual number of drinks per day, Table 4, kappa = 0.507). This makes sense because the referent 30-day period for drinking was different.

**Table 3:** Comparison of self-reported cigarette smoking between PMRP and PhenX

**PMRP**	**PhenX**			
**Smoked at least 100 cigarettes in entire life**				
	No	Yes	Combined	
	N (%)	N (%)	N (%)	
No	**1151 (52.1)**	16 (0.7)	1167 (52.9)	
Yes	88 (4.0)	**953 (53.2)**	1041 (47.1)	
Combined	1239 (56.1)	969 (43.9)	2208 (100.0)	
Kappa = 0.905 (95% CL=0.887, 0.923)				
**Frequency of current smoking PMRP enrollment and PhenX follow-up**				
	Every day	Some days	Not at all	Combined
	N (%)	N (%)	N (%)	N (%)
Every day	**60 (2.7)**	12 (0.5)	39 (1.8)	111 (5.0)
Some days	8 (0.4)	**10 (0.5)**	23 (1.0)	41 (1.9)
Not at all	13 (0.6)	9 (0.4)	**2027 (92.1)**	2049 (100.0)
Combined	81 (3.7)	31 (1.4)	2089 (94.9)	2201 (100.0)
Kappa = 0.673 (0.604, 0.741)				

The numbers are the actual counts (and percent) of people responding “yes” or “no” on the two questionnaires about whether they had ever smoked 100 cigarettes in their lifetime and whether they were currently smoking. Agreement (no/no or yes/yes) is noted in bold.

**Table 4:** Comparison of self-reported alcohol intake between PMRP and PhenX

**PhenX**					
	**<1**	**1-2**	**3-4**	**5-7**	**Combined**
**PMRP**	**N (%)**	**N (%)**	**N (%)**	**N (%)**	**N (%)**
**Number of days in the past 30 days that respondent drank one or more drinks**					
<1	**1204 (54.8)**	89 (4.0)	22 (1.0)	9 (0.4)	1324 (60.2)
1-2	224 (10.2)	**141 (6.4)**	41 (1.9)	16 (0.7)	422 (19.2)
3-4	63 (2.9)	66 (3.0)	**52 (2.4)**	35 (1.6)	216 (9.8)
5-7	42 (1.9)	28 (1.3)	33 (1.5)	**133 (6.1)**	236 (10.7)
Combined	1533 (69.7)	324 (14.7)	148 (6.7)	193 (8.8)	2198 (100.0)
Kappa = 0.666 (95% CL=0.627, 0.704)					
	**<1**	**1-2**	**3-4**	**5 or more**	**Combined**
**PMRP**	**N (%)**	**N (%)**	**N (%)**	**N (%)**	**N (%)**
**Usual number of drinks per day**					
<1	**502 (24.9)**	96 (4.8)	7 (0.3)	6 (0.3)	611 (30.4)
1-2	231 (11.5)	**921 (45.8)**	47 (2.3)	21 (1.0)	1220 (60.6)
3-4	15 (0.7)	67 (3.3)	**51 (2.5)**	11 (0.5)	144 (7.2)
5 or more	6 (0.3)	8 (0.4)	10 (0.5)	**14 (0.7)**	38 (1.9)
Combined	754 (37.4)	1092 (54.2)	115 (5.7)	52 (2.6)	2013 (100.0)
Kappa = 0.507 (95% CL=0.445, 0.5688)					

The numbers are the actual counts (and percent) of people reporting on the two questionnaires the number of days that they had an alcoholic drink in the previous 30 days how drinks they had on a typical days in the previous 30 days. Agreement is noted in bold.

Table 5 summarizes the comparison of PhenX measures with PMRP questionnaire and Marshfield Clinic medical record data. The PhenX questionnaire included a question about whether the respondent had ever been told by a physician that they had a stroke, as well as a series of questions about symptoms associated with stroke. The PhenX responses were compared with diagnosis codes for stroke and transient ischemic attack (TIA) from Marshfield Clinic electronic medical records. 139 (6.2%) of subjects indicated on the PhenX questionnaire that they had been told they had a stroke. Of them, only 15 (10.8%) had no electronic indication of a prior stroke or TIA. The agreement for no report of physician-reported stroke on the PhenX questionnaire with no stroke or TIA codes appearing in the Marshfield Clinic EMR was 99.2%. The negative predictive value of self-reported physician-diagnosed stroke (1875/1912, 98.1% when no TIA code was found) was found to be higher than the positive predictive value (92/113, 81.4% when TIA code was present in the EMR).

**Table 5:** Comparison of stroke history as reported on PhenX survey and as in medical records

**PhenX Physician-reported stroke**				
		**No**	**Yes**	
**TIA code?**	**Stroke codes?**	**-------------**	**-------------**	
		**N (row %)**	**N (row)**	**Kappa statistic (95% CL)**
No	None	1875 (99.2)	15 (0.8)	
	1 only	23 (95.8)	1 (4.2)	
	2 or more	14 (58.3)	10 (41.7)	0.285 (0.148, 0.422)
Yes	None	155 (88.1)	21 (11.9)	
	1 only	14 (63.6)	8 (36.4)	
	2 or more	30 (26.3)	84 (73.7)	0.568 (0.476, 0.660)

Table 6 includes data to compare self-reported family history of myocardial infarction between PhenX and PMRP. The simple kappa statistic for the agreement was 0.352 (95% CL = 0.317, 0.386). In the PMRP enrollment questionnaire, subjects were asked if they had two or more first degree relatives, including themselves, who had ever had heart attack or angina. 589 of the subjects in the current study reported a family history of heart attack or angina on the PMRP questionnaire. 1108 of subjects reported in the PhenX questionnaire that at least one of their first degree relatives had a myocardial infarction. It is difficult to compare the two responses because the questions were asked differently, included different people (self in the PMRP questionnaire), and there was a time gap of an average eight years between administration of the two questionnaires.

**Table 6:** Family history* of heart attack as reported on PhenX survey and of heart attack/**angina as reported in PMRP**

**PhenX family history of MI?**				**PMRP family history of MI**/**Angina**			
**Yes**		**No**		**Yes**		**No**	
**N**	**%**	**N**	**%**	**N**	**%**	**N**	**%**
1108	52.8	991	47.2	589	28.1	1510	71.9
Kappa=0.352 (95% CL=0.317, 0.386)							

*Family history of MI or angina in first degree relatives.

Table 7 summarizes age- and sex-specific prevalence of major depressive disorder from the PhenX measure and previously published data [7-9] using the WHO CIDI-SF (the selected PhenX measure of depression). All of the stratum-specific 95% confidence limits overlap and show that 31% of women aged 50–64 reported symptoms associated with a major depressive episode.

**Table 7:** Age and sex-specific distribution of PhenX WHO CIDI-SF major depression episode (MDE) and comparison with published data

**Male**	**Female**			
**Study**	**Age 50-64**	**Age 65+**	**Age 50-64**	**Age 65+**
	**MDE% (9.5% CL)**	**MDE% (95% CL)**	**MDE% (95% CL)**	**MDE% (95% CL)**
PhenX	14.2 (9.5, 18.8)	8.8 (6.7, 11.0)	31.3 (26.6, 36.1)	11.9 (9.8, 14.0)
NCR-R*	16.2 (13.5, 18.9)	5.3 (2.9, 7.7)	24.6 (21.7, 27.5)	13.0 (10.5. 15.5)

*National Comorbidity Surveys Replication, Kessler et al. 2010 [8].

Current symptom severity for respondents reporting lifetime major depression symptoms was moderate or greater in 4.9% of respondents while 75.6% of participants reported no current symptoms of depression (Table 8).

**Table 8:** QIDS-SR depression symptom severity reported on the PhenX survey

**None**	**Mild**	**Moderate**	**Severe**	**Very severe**
**N (%)**	**N (%)**	**N (%)**	**N (%)**	**N (%)**
1582 (75.6)	409 (19.5)	87 (4.2)	12 (0.6)	3 (0.1)

## Discussion

To our knowledge, this is one of the first large-scale implementations of PhenX Toolkit measures since their release. The use of standardized tools is vital to discovery efforts in the field of medical genomics. We quickly discovered in the eMERGE network that larger sample sizes than were originally anticipated were needed for straight GWAS analyses, in part because of different technologies and phenotype definitions used across the network [3]. Gene/environment analyses are further compromised when standardized tools are not used because data cannot be reliably merged across studies to allow for necessary validation or increased sample sizes for meta analyses that yield statistically significant results. Use and incorporation of PhenX data into dbGaP along with GWAS data will facilitate large-scale gene/environment studies and we support these efforts. The PhenX data have been submitted to dbGaP (dbGaP study accession: phs000170.v1.p1) for the current study to be merged with other phenotypic data and GWAS genotypes already available in dbGaP to the research community. The dbGaP website contains information about how to access data (http://www.ncbi.nlm.nih.gov/gap).

Many of the items that we selected from the Toolkit were intended for interviewer-administration. We selected items based on content, not mode of administration and had to remove interviewer instructions prior to administration. With feedback from the PhenX RISING network, the Toolkit has been amended to allow researchers to select a self-administered option. After completion of formatting to allow self-administration, we found the PhenX Toolkit easy to use with minimal queries from participants about how to complete the forms. Most questions were related to the Family Health History section for heart attack or myocardial infarction because of difficulty in understanding the table format. Some people needed clarification related to the type of dwelling they lived in fitting their home into one of the category options listed. A few queries were related to depression, stroke follow-up questions and sun exposure. The data are being mapped in dbGaP to the PhenX Toolkit measures to allow other researchers to combine PhenX data across studies to increase statistical power for gene/environment studies.

Observed differences between the PhenX and PMRP were expected for some variables, such as age, because of the time difference between enrollment into PMRP and completion of the PhenX questionnaire. The lower mean weight and concomitant BMI in PhenX would not be expected because average weight generally increases as a population ages. However, the mode of data collection was different. At the time of enrollment into PMRP, participants had standardized measurements of height and weight from which BMI was calculated [4]. For PhenX, weight and height were self-reported. A systematic review of studies comparing self-reported and measured height and weight found a trend of under-reporting of weight and over-reporting of height which was inconsistent [10], and which would explain the lower mean weight observed in the PhenX questionnaire when compared with the direct measurement at enrollment into PMRP. Specific instructions within the PhenX Toolkit warn researchers that “Self-reported weight values are considered to be less accurate. Self-reported weight is subject to error and is used when measured weight cannot be obtained”. Because of the inconsistency in the inaccuracy of self-report, it is not possible to create rules to adjust self-reported weight or to assume the relative position of weight in a population is constant. Our data support the PhenX Toolkit cautionary note to only use self-reported weight when it is not possible to obtain a measured weight.

There was a large difference in self-reported family history of heart attack between the two questionnaires in the current study (52.8% versus 28.1%) and there are several potential reasons for this difference. First, the time difference between administration of the two questionnaires provided more opportunity for first degree relatives to experience a heart attack by the time of the PhenX questionnaire and in fact the rate was higher in that survey. Second, the questions were not asked identically. The PMRP question included both angina and heart attack. Accuracy of self-reported family history has been shown to vary by personal health history [11].

The positive predictive value of self-reported physician-diagnosed stroke was found to be lower than the negative predictive-value in the present study (81.4% versus 98.1%). A study conducted in Olmstead County, Minnesota revealed positive and negative predictive values for stroke including TIA of 67.4% and 99.2% respectively, with higher levels of agreement observed in older ages, women, and more educated individuals [12]. In addition to the difference in disease definition, mode of administration may have led to observed differences. The PhenX stroke protocol was intended to be interviewer-administered and was self-administered in the current study and the gold standard for the current study was physician assessment. Consideration should be given to being more specific with the PhenX question so that respondents understand the difference between TIA and stroke because they are not identical terms.

Data for direct validation of the major depressive episode (MDE) PhenX questions were not available but a comparison of the rates documented in PMRP with the PhenX Toolkit revealed markedly similar MDE rates with previously published age- and gender-specific rates from the WHO World Mental Health Survey Initiative [8,9]. This lends external validity to the results.

## Conclusions

In conclusion, we demonstrated the ease and utility of the PhenX Toolkit to quantify exposures that can be used to facilitate gene/environment analyses. Future studies will leverage available GWAS data for this cohort of participants.

## Competing interests

The authors declare that they have no competing interests.

## Authors’ contributions

CAM designed the study, secured funding, and prepared the initial draft of the manuscript. RB assisted in study design conducted the statistical analyses. CMR assisted in the data collection and data analyses. CJB assisted in data collection and interpretation. TK assisted in data collection and interpretation. MB assisted in study coordination and interpretation of the data. MDR assisted in study design and data interpretation. All authors read and approved the final manuscript.

## Pre-publication history

The pre-publication history for this paper can be accessed here:

http://www.biomedcentral.com/1755-8794/7/3/prepub
